# Validating laboratory defined chronic kidney disease in the electronic health record for patients in primary care

**DOI:** 10.1186/s12882-018-1156-2

**Published:** 2019-01-03

**Authors:** Martin Frigaard, Anna Rubinsky, Lo Lowell, Anna Malkina, Leah Karliner, Michael Kohn, Carmen A Peralta

**Affiliations:** 1 Kidney health research collaborative (KHRC), University of California, 4150 Clement St. Building 2, Room 145, San Francisco, CA 94121 USA; 2 Nephrology and Hypertension at Parnassus, University of California San Francisco, 400 Parnassus Ave, San Francisco, CA 94122 USA

**Keywords:** Chronic kidney disease, Electronic health record phenotype, Validation

## Abstract

**Background:**

Electronic health record (EHR) data is increasingly used to identify patients with chronic kidney disease (CKD). EHR queries used to capture CKD status, identify comorbid conditions, measure awareness by providers, and track adherence to guideline-concordant processes of care have not been validated.

**Methods:**

We extracted EHR data for primary-care patients with two eGFRcreat 15-59 mL/min/1.73 m^2 at least 90 days apart. Two nephrologists manually reviewed a random sample of 50 charts to determine CKD status, associated comorbidities, and physician awareness of CKD. We also assessed the documentation of a CKD diagnosis with guideline-driven care.

**Results:**

Complete data were available on 1767 patients with query-defined CKD of whom 822 (47%) had a CKD diagnosis in their chart. Manual chart review confirmed the CKD diagnosis in 34 or 50 (68%) patients. Agreement between the reviewers and the EHR diagnoses on the presence of comorbidities was good (κ > 0.70, *p* < 0.05), except for congestive heart failure, (κ = 0.45, *p* < 0.05). Reviewers felt the providers were aware of CKD in 23 of 34 (68%) of the confirmed CKD cases. A CKD diagnosis was associated with higher odds of guideline-driven care including CKD-specific laboratory tests and prescriptions for statins. After adjustment, CKD diagnosis documentation was not significantly associated with ACE/ARB prescription.

**Conclusions:**

Identifying CKD status by historical eGFRs overestimates disease prevalence. A CKD diagnosis in the patient chart was a reasonable surrogate for provider awareness of disease status, but CKD awareness remains relatively low. CKD in the patient chart was associated with higher rates of albuminuria testing and use of statins, but not use of ACE/ARB.

**Electronic supplementary material:**

The online version of this article (10.1186/s12882-018-1156-2) contains supplementary material, which is available to authorized users.

## Background

Optimizing primary care clinical management of chronic kidney disease (CKD) remains a critical step to reduce overall disease burden [1]. The widespread adoption of electronic health records (EHR) in the United States has garnered excitement about accomplishing this goal by transforming the clinical environment into a “learning health care system” [2–4]. In this context, a learning healthcare system is one that can collect and store accurate information for each patient, and in turn, use these data to inform and support improvements in clinical care [5, 6]. Pragmatic randomized trials using the EHR to identify individuals with CKD and quantify the gaps in their care, followed by interventions to improve outcomes, can rigorously test the promise of these learning healthcare systems. In fact, the National Institutes of Health (NIH) has published several requests for applications explicitly focused on design and evaluation of information technology-based tools and interventions to improve CKD care [7, 8].

Necessary pre-requisites to the successful design, implementation, interpretation and dissemination of the results of such pragmatic trials include data accuracy and adequate quantification of gaps in care [9]. Specifically, prior to the design and implementation of EHR-based pragmatic trials, investigators must be confident in the ability of the existing data to identify patients with CKD who are at risk for complications, deliver an intervention with a high probability of improving care, and ascertain relevant outcomes [10]. Accurate phenotyping (i.e. the ability to accurately classify disease status) is important because misclassification can introduce bias and limit interpretation of results [11, 12]. The few pragmatic studies that have used EHR data designed to improve care in individuals with CKD have not reported detailed methodology regarding phenotyping process or ascertainment of the clinical gaps prior to design and implementation [13–17]. Prior registry studies that have validated CKD diagnostic codes to classify CKD status were primarily conducted in inpatient hospital settings, and even fewer have used clinician chart review as a gold standard [18, 19]. One of the largest validated EHR-based CKD registries did not specifically focus on patients actively seen in primary care practices [20]. Investigators must also be able to quantify the clinical practice gap(s) and the potential for resolution of that gap(s) to improve outcomes (i.e. “actionable gap”) [21]. For example, the lack of PCP awareness of CKD has been cited as a major barrier to optimal CKD care, but the degree and importance of this lack of awareness may vary greatly by setting [22, 23]. Thus we see a gap in knowledge between the specific methods used to identify CKD patients in the EHR and the evaluation of the appropriate guideline-driven care practices among the identified patients.

In this methodologic study, we set out to investigate the use of historical laboratory data to identify a cohort of primary care patients with CKD, and, in a subset of the cohort, have clinical nephrologists confirm a CKD diagnosis and the presence or absence of comorbidities known to influence CKD via manual chart review. Additionally, we evaluated the prevalence and usefulness of CKD documentation in the EHR problem list as a surrogate for primary care provider (PCP) awareness of CKD status by assessing its association with guideline-driven care [24].

## Methods

### Setting and cohort development

We used data from patients regularly seen in primary care at the University of California San Francisco (UCSF) General Internal Medicine (GIM) practices from 2014 to 2016. This practice provides comprehensive primary care internal medicine services for a diverse population of adults with a mixture of public and private insurance. UCSF medical professionals use Epic (Epic Systems Corporation) for appointment scheduling, laboratory and medication orders, recording progress notes, documenting diagnoses, prescription management, and communication between providers and patients [25]. The problem list contains the current and active diagnoses for each patient. The UCSF Institutional Review Board approved the data and methods for this project.

**Fig. 1:**
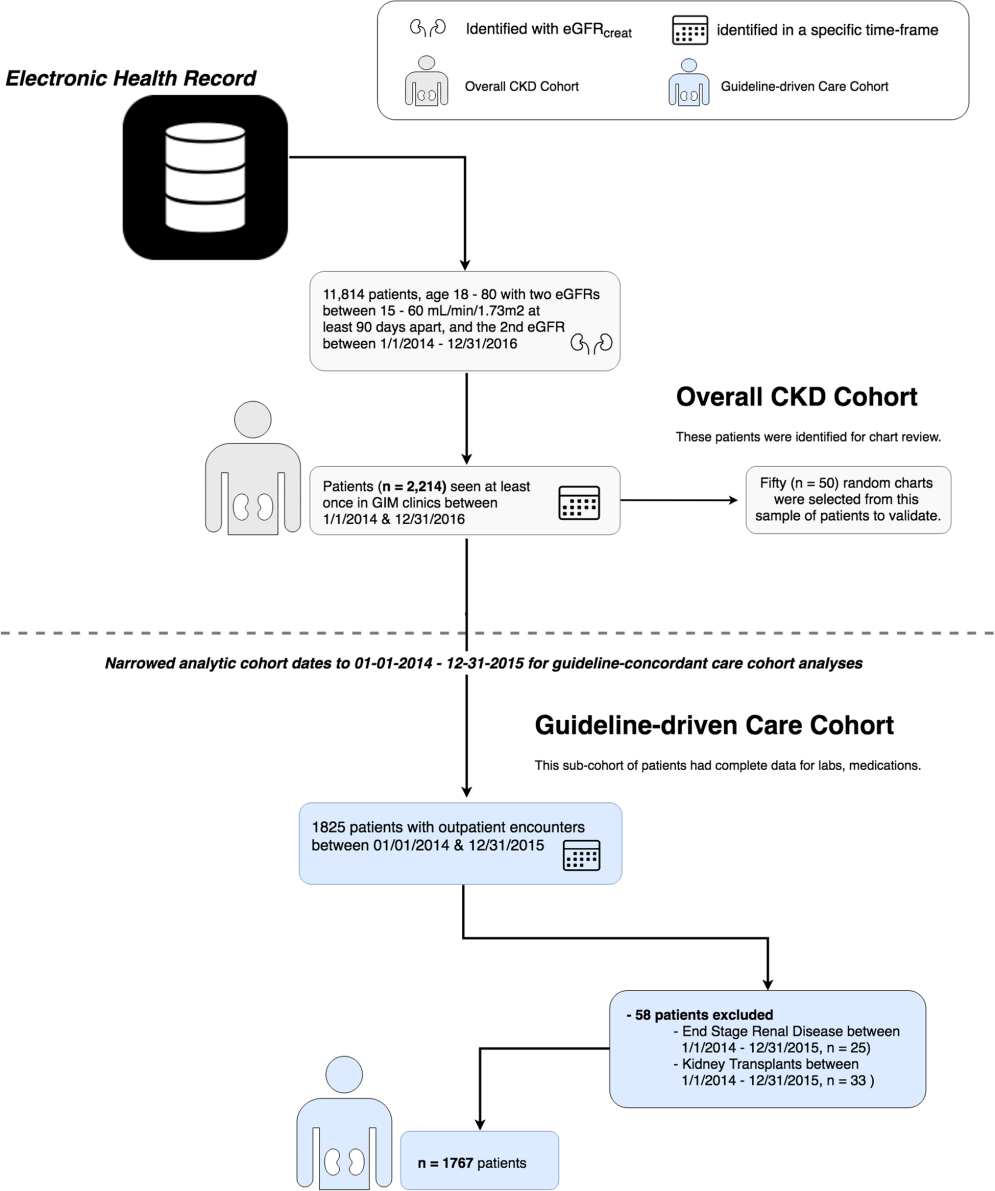
Patient cohort electronic health record data extraction

We’ve outlined the data extraction steps in Fig. 1. Our goal was to identify a cohort of patients with CKD defined by historical laboratory values and who also had regular follow-up in primary care. We define this as the *overall CKD cohort*, and it was created by identifying patients aged 18–80 with at least two serum creatinine measurements at least 90 days apart. We calculated each patient’s eGFR_creat_ using the CKD Epi equation when not automatically reported [26]. To enter the overall CKD cohort, we required that patients have two eGFR_creat_ between 15 and 59.999 ml/min per 1.73m^2^ at least 90 days apart, with their second “qualifying” eGFR_creat_ in the period from 1/1/2014 and 12/31/2016. To include patients with regular follow-up, we also limited the overall CKD cohort to patients with at least one primary care encounter in the UCSF GIM practice between 1/1/2014 and 12/31/2016. We selected 50 random charts from the overall CKD cohort for validation as described below (Measures).

We then derived a sub-cohort of patients for whom we had complete data on laboratory measurements, medications, and outpatient encounters (the guideline-concordant care cohort in Fig. 1). To ensure a complete set of data, we further restricted the guideline-concordant care cohort to patients who had their second “qualifying” eGFRcreat between 1/1/2014 and 12/31/2015. We also excluded patients from the guideline-concordant care cohort if they had problem list documentation of 1) receiving a kidney transplant or 2) having end-stage renal disease (ESRD) during the study period.

### Measures

#### CKD diagnosis: Problem list and nephrologist confirmation by chart review

We defined “Problem List CKD” as the presence of an EHR problem list diagnosis of CKD in each cohort using the following International Statistical Classification of Diseases, Ninth Revision, Clinical Modification (ICD9) codes: 585, 585.1, 585.2, 585.3, 585.4, 585.4, 585.5, 585.6, 585.9. Epic© records patient diagnoses with an internal identifier to ensure a physician can accurately capture the patient’s condition according to the current ICD code, while maintaining backwards compatibility with previous diagnosis codes.

We defined ‘nephrologist confirmed CKD’ via chart review of a random sample of 50 charts in the overall CKD cohort. Two nephrologists (LL & AM) were asked to review the 50 charts only using data from January 1, 2013 to December 31, 2016. They were instructed to examine labs, echo, imaging, notes, medications, and vital signs to determine if each patient had the following conditions: hypertension, chronic kidney disease, diabetes, coronary artery disease, peripheral vascular disease, congestive heart failure, or cerebrovascular disease (defined as a history of stroke or TIA). The responses were binary (Yes/No), and in the event of a disagreement, a third nephrologist (CP) served as the tie-breaker.

Since we were also interested in determining the validity of using a CKD diagnosis on the problem list as a proxy for CKD awareness by the primary care provider, the reviewers were also asked to read the primary care physician’s notes to determine if they thought the PCP was, “aware that the patient has CKD.” We refer to this measure as “CKD awareness”. All three reviewing clinicians used a combination of the guideline-concordant definition of CKD with clinical judgment to determine each patient’s CKD status [24].

#### Demographics and comorbidities

We extracted demographic characteristics from the EHR including age (at the time of the patient’s second ‘qualifying’ eGFR_creat_), sex, ethnicity (Hispanic or Latino, Not Hispanic or Latino, Not Specified), and race (African American, Asian, White, and Other/Unknown). Patients who indicated multiple races and included Black or African American, were considered Black or African American due to the impact on eGFR_creat_. We also extracted five comorbid conditions from the EHR: cerebrovascular disease, congestive heart failure, coronary artery disease, diabetes mellitus, and hypertension. We defined the presence of CKD and each comorbid condition with an ICD9 documented in the patient’s (Additional file 1: Appendix Table S1) problem list or associated with an outpatient encounter.

#### Outcomes: Guideline-concordant processes of care

We selected the processes of care outlined in the 2012 Kidney Disease, Improving Global Outcomes (KDIGO) guidelines [24]. These included any laboratory test ordered for albuminuria (albumin-to-creatinine ratio), mineral metabolism disorder (serum phosphate, 25-hydroxyvitamin D, and intact parathyroid hormone), any prescription orders for angiotensin-converting enzyme inhibitors or angiotensin receptor blockers (ACEi/ARB), and any active prescription for statins between 1/1/2014 and 12/31/2015. We also extracted the number of nephrology office visits and nephrology consultations between 1/1/2014 and 12/31/2015.

### Statistical analyses

#### Chart review analyses

We began by comparing characteristics of the 50 patients randomly selected for chart review against the overall cohort. We initially evaluated differences in age, race, ethnicity, eGFR_creat_, and CKD on the problem list. We then determined the prevalence CKD on the problem list in the overall sample and the 50 random charts. Next--among the 50 randomly selected patients’ charts--we compared characteristics of patients with CKD confirmed by clinician chart review vs. unconfirmed. We tested differences in patient characteristics by eGFRcreat category using one-way analysis-of-variance (ANOVA) for continuous variables and Chi-Square or Fisher exact tests for categorical variables. If there was any question about the normality of a continuous variable’s distribution, we used a nonparametric equivalent test to confirm the parametric results. We then compared prevalence of the comorbidities (cerebrovascular disease, congestive heart failure, coronary artery disease, diabetes mellitus, and hypertension) by ICD9 codes vs. clinician chart review. We also calculated the sensitivity, specificity, negative and positive predictive values for these five comorbid conditions—again comparing the administrative codes against the clinician chart review. Because this cohort was comprised entirely of patients with eGFR_creat_ defined CKD, we were only able to capture the prevalence for CKD and CKD awareness.

#### Guideline-concordant CKD care analyses

In the second part of our analyses, we used data from the guideline-concordant care sub-cohort described above. We first compared clinical and demographic characteristics by eGFR_creat_ level, and we estimated the proportion of patients who had CKD on the problem list overall and by eGFR_creat_ category. We then tested for associations of problem list CKD documentation with each process of care measure individually, using logistic regression to model the odds of receipt of guideline-concordant care. We initially adjusted for age, sex, race, and ethnicity in model one. In model two, we additionally adjusted for degree of CKD by adding the patient’s second ‘qualifying’ eGFR_creat_ (15.0–59.999 ml/min per 1.73m^2^), and for comorbidities by adding indicator variables for the presence or absence of each problem list comorbidity diagnosis (diabetes mellitus, cerebrovascular disease, congestive heart failure, hypertension, coronary artery disease). We also tested whether the association of problem list CKD documentation and any orders for albuminuria testing, ACEi/ARB prescription, or statin prescription differed between patients seen by a nephrologist vs. those followed only in primary care. Specifically, we repeated models one and two stratified by whether the patient had received a nephrology consultation, and tested for an interaction. Statistical significance of our findings was evaluated using an alpha level of *P* < 0.05. All analyses were conducted using R (version 3.4.3).

## Results

### Overall CKD cohort and chart review

For the overall CKD cohort, we identified 2214 UCSF primary care patients who met eGFR_creat_ criteria for CKD and were eligible for the chart review (Fig. 1). Comparison of characteristics for those randomly sampled for the chart review (*n* = 50) to the overall CKD cohort demonstrated no significant difference in demographic or clinical characteristics (Table 1). Overall, the mean age was 68 (IQR 61–75), mean eGFR_creat_ was 52 (IQR 44–57). Most (81%) had hypertension, and a large proportion (41%) had diabetes. A total of 943 (44%) had CKD listed on their problem list.

**Table 1:** Characteristics of primary care patients with CKD based eGFR^†^

	Extracted cohort	Charts for review	
	*n* = 2164	*n* = 50	
	*Median (IQR) or N (%)*		*p*-value
Demographic characteristics			
Age*	68 (61–75)	66 (62–74)	0.81
Male	1122 (52)	26 (52)	1.00
Ethnicity, *N* (%)			
Not Hispanic or Latino	1957 (90)	46 (92)	0.64
Hispanic or Latino	169 (8)	4 (8)	
Unknown/Declined	38 (2)	0 (.0)	
Race, *N* (%)			
Other/Unknown	322 (15)	12 (24)	0.35
Asian	525 (24)	10 (20)	
African American	309 (14)	7 (14)	
White	1008 (47)	21 (42)	
Patient 2nd eGFR			
eGFR	52 (44–57)	52 (40–57)	0.93
eGFR categories			
< 30	182 (8)	6 (12)	0.67
30–44	399 (18)	9 (18)	
45–60	1583 (73)	35 (70)	
Problem list CKD			
Yes	943 (44)	24 (48)	0.63
Comorbid conditions			
Coronary artery disease	487 (23)	9 (18)	0.56
Congestive heart failure	228 (11)	7 (14)	0.58
Cerebrovascular disease	287 (13)	5 (10)	0.64
Diabetes mellitus	880 (41)	22 (44)	0.74
Hyptertension	1754 (81)	39 (78)	0.72

^†^Characteristics of patients included in process of care analyses, base on data from the Overall CKD Cohort (see Fig. 1)

*Age at earliest second “qualifying” eGFR

Among the 50 patients randomly sampled for chart review, 34 (68%) had nephrologist confirmed CKD. Those identified as having CKD by historical eGFR_creat_ but not confirmed by chart review were more likely to be female, White and have higher eGFR_creat_ levels compared to those with CKD confirmed by the nephrologists’ chart review. Those for whom CKD was not confirmed were less likely to have diagnoses of hypertension or diabetes. All 16 individuals with CKD not confirmed had eGFR_creat_ greater than 45 ml/min/1.73m^2^ (Table 2). We also found that only 1 of the 16 individuals with CKD not confirmed had CKD listed on the problem list, while 68% of those with nephrologist confirmed CKD had CKD listed on their problem list.

**Table 2:** Characteristics of 50 patients categorized by clinician chart review confirmation^†^

	No CKD by Clinician Chart Review	CKD by Clinician Chart Review
	*n* = 16	*n* =34
	*Median (IQR) or N (%)*	
Demographic characteristics		
Age*	68 (62–74)	66 (64–74)
Female	11 (69)	13 (38)
Ethnicity		
Not Hispanic or Latino	16 (100)	30 (88)
Hispanic or Latino	0 –	4 (12)
Unknown/Declined	0 –	0 –
Race		
Other	1 (6)	11 (32)
Asian	3 (19)	7 (21)
African American	2 (12)	5 (15)
White	10 (62)	11 (32)
Patient 2nd eGFR		
eGFR	57 (52–59)	47 (36–55)
eGFR categories		
< 30	0 –	6 (18)
30–44	0 –	9 (26)
45–60	16 (100)	19 (56)
Problem list CKD		
Yes	1 (6)	23 (68)
Comorbid conditions, *N* (%)		
Coronary artery disease	3 (19)	6 (18)
Congestive heart failure	1 (6)	6 (18)
Cerebrovascular disease	2 (12)	3 (9)
Diabetes mellitus	2 (12)	20 (59)
Hyptertension	8 (50)	31 (91)

^†^Characteristics of patients included in process of care analyses, base on data from the Overall CKD Cohort (see Fig. 1)

*Age at earliest second “qualifying” eGFR

By examining notes, the nephrologists determined that 23 (68%) of the 34 patients with confirmed CKD also had primary care providers who were aware of the patient’s CKD. Among the 23 patients for whom the nephrologists believed the PCP was aware of the patient’s CKD, 21 (91%) had CKD documented on their problem list.

When we evaluated phenotyping of comorbid condition, we found good agreement between ICD-9 diagnoses and chart review for most comorbid conditions, except congestive heart failure, which was only in moderate agreement (Table 3). The sensitivity was lowest for congestive heart failure and coronary artery disease, and highest for cerebrovascular disease and diabetes mellitus. Specificity values were high, with the lowest values for congestive heart failure and diabetes mellitus.

**Table 3:** Agreement between administrative (ICD-9) diagnosis codes and manual chart review^a^

Comorbidity	Prevalence (Chart Review)	Prevalence (EHR)	Sensitivity^a^	Specificity^a^	Kappa Statistic	*p*-value ‡
Cerebrovascular disease	10%	8%	100%	98%	0.88	< 0.001
Congestive heart failure	14%	16%	50%	93%	0.45	0.001
Coronary artery disease	18%	22%	82%	100%	0.88	< 0.001
Diabetes mellitus	44%	42%	95%	93%	0.88	< 0.001
Hypertension	78%	86%	91%	100%	0.73	< 0.001

^a^Calculated for ICD-9 diagnosis of each condition compared to chart review (including clinical notes, ICD-9 diagnoses, laboratory results etc.)

^‡^*p*-values are calculated from Cohen’s Kappa test statistic as an index of interrater agreement between 2 raters on categorical data

### Guideline-driven care CKD sub-cohort

Of the overall CKD cohort, we had complete data for 1825 persons, and these patients had an earliest second ‘qualifying’ eGFR_creat_ on or before 12/31/2015. After we excluded and additional 58 patients due to either end-stage renal disease (ESRD) or a kidney transplant on their problem list, a total of 1767 were included in the guideline-driven care CKD sub-cohort. The characteristics of these patients are presented in Additional file 2: Appendix Table S2, stratified by eGFR_creat_ category. Among 1767 patients, a total of 822 (47%) had a CKD diagnosis on their problem list. The prevalence of CKD on the problem list increased with increasing severity of CKD (Additional file 2: Appendix Table S2). Specifically, among individuals with eGFR_creat_ < 30 ml/min/1.73m^2^, 79% had CKD listed, compared with 68% with eGFR_creat_ 30–45 ml/min/1.73m^2^ and 37% of patients with an eGFR_creat_ 45–60 ml/min/1.73m^2^ (*p* < 0001).

In the logistic regression models adjusted for age, sex, race, and ethnicity, we found that patients with CKD documented in their problem list had more than two-fold higher odds of having a test order for albuminuria, phosphorus and vitamin D, and nearly seven-fold higher odds for having a test order for parathyroid hormone compared to patients with no documentation (Table 4). Patients with CKD documented in their problem list had nearly two-fold higher odds of having a prescription for an ACEi/ARB or for a statin compared to patients with no documentation. After adjustment for comorbidities and CKD severity, these associations were somewhat attenuated, but remained statistically significant, except for ACEi/ARB prescriptions. The associations of CKD documentation in the problem list with any order for albuminuria, any prescription for an ACEi/ARB, and any prescription for a statin were not materially different after stratification by the presence or absence of a nephrology consultation (*p* > 0.05). See Additional file 3: Appendix Table S3 and Additional file 4: Appendix Table S4.

**Table 4:** Associations between listing of CKD on problem list with guideline-driven processes of care^a^

	Outcome	Model 1	Model 2
Problem List CKD	*Albuminuria test ordered*	Odds Ratio	Odds Ratio
	*N* (%)	(95% C.I.)	(95% C.I.)
No = 932	288 (30%)	*Ref*	*Ref*
Yes = 679	472 (57%)	2.94 (2.39–3.62) †	2.83 (2.17–3.70) †
Problem List CKD	*Parathyroid hormone test ordered*		
No = 932	109 (12%)	*Ref*	*Ref*
Yes = 679	400 (49%)	6.94 (5.40–8.92) †	5.03 (3.87–6.54) †
Problem List CKD	*Phosphorus test ordered*		
No = 932	484 (51%)	*Ref*	*Ref*
Yes = 679	601 (73%)	2.24 (1.81–2.77) †	1.60 (1.27–2.01) †
Problem List CKD	*Vitamin D test ordered*		
No = 932	446 47%	*Ref*	*Ref*
Yes = 679	573 70%	2.66 (2.16–3.27) †	2.35 (1.88–2.93) †
Problem List CKD	*ACE/ARB prescription*		
No = 932	543 (57%)	*Ref*	*Ref*
Yes = 679	570 (69%)	1.63 (1.32–2.01) †	1.21 (0.94–1.55) †
Problem List CKD	*Statin prescription*		
No = 932	565 (60%)	*Ref*	*Ref*
Yes = 679	600 (73%)	1.91 (1.53–2.37) †	1.56 (1.21–2.02) †

^a^Characteristics of patients included in process of care analyes, based on data from 01/01/2014 to 12/31/2015

Model 1 includes age, sex, race, and ethnicity

Model 2 adds each patient’s earliest 2nd qualifying eGFR (15–59.999 mL/min/1.73 m2) and comorbidities (diabetes mellitus, cerebrovascular disease, congestive heart failure, hypertension, and coronary artery disease)

^†^ = *p* < 0.05

## Discussion

In this methodologic study, we show findings with important implications for design and implementation of future pragmatic studies that leverage the EHR to deploy interventions to improve management of individuals with CKD in primary care. First, we found that using a CKD definition based on historical eGFR_creat_ values only may be too sensitive, since we found that CKD status was confirmed by nephrologist chart review in only 68% of cases. Female gender, White race and higher eGFR_creat_ were more common among persons with CKD that was not ultimately confirmed by chart review. We also show that CKD on the problem list is a relatively good surrogate for PCP awareness, as it has high concordance with both CKD status and PCP awareness as ascertained by nephrologist chart review. In the primary care practice included in this study, providers have only moderate rates of CKD awareness (44%), defined as CKD present on the problem list. However, awareness increased significantly with severity of CKD. Importantly, CKD awareness was significantly associated with higher odds of guideline-concordant CKD testing, and the use of ACEi/ARB and statins.

Eight prior studies have reported misclassified CKD diagnoses from administrative data [18–20, 23]. However, five of these studies were conducted in an inpatient hospital setting or used patients who were hospitalized [18]. Only three of these studies sampled from outpatient primary care settings and included expert clinician chart review as a gold standard [19, 20]. We identified only one study that focused on patients who were receiving active and ongoing outpatient primary care [23]. Narrowing the focus to these patients is critical because these are the individuals who are most likely to be included in intervention studies. Recently, the eMERGE consortium published an algorithm that maximizes sensitivity and specificity of CKD associated with hypertension and diabetes, compared with chart review [20]. Appropriate implementation of the algorithm requires data handling procedures that natural language processing of text, which are unlikely to be feasible for most primary care practices [20]. Our study adds value to the literature as it shows that CKD ascertained only by two historical eGFR_creat_ values < 60 ml/min/1.73m^2^ at least 90 days apart may be too sensitive when the goal is to deploy interventions for those individuals with CKD at highest risk for complications who are most likely to benefit from interventions. Our finding that almost a third of patients identified as having CKD by eGFR_creat_ did not have CKD confirmed by expert physicians has important implications because it is likely that future pragmatic trials will need to include steps to re-test and further risk stratify patients (e.g., with albuminuria and cystatin C testing) to confirm CKD before deploying interventions.

In our study, combining problem list and encounter diagnoses to identify the comorbidities most likely to be relevant in studies of CKD patients correlated well with expert physician chart review. One important exception was presence of heart failure, a finding similar to prior studies showing that presence of heart failure correlates relatively poorly with administrative codes alone [27]. This suggests that, if researchers aim to include or exclude individuals with certain comorbidities from intervention studies, administrative codes and problem list are suitable for most of the conditions we tested, but not for heart failure where additional variables or chart review will be required.

We also found that CKD listed on the problem list may be a good surrogate for PCP awareness of this diagnosis. Low PCP awareness of CKD has been cited as one of the major barriers to improve kidney care in the U.S. [28]. Yet strategies to ascertain the degree of awareness are limited. We showed that, in this setting, CKD documented on the problem list had high concordance with CKD awareness when ascertained by expert chart review. Importantly, we also confirmed findings from prior studies that CKD awareness by PCP remains limited [22, 23, 28]. While only 44% of patients had CKD listed, which is higher than some previously reported estimates, CKD awareness did significantly increase with severity of CKD [19, 22, 23]. This suggests that CKD documentation may be a useful additional variable to increase the specificity of EHR data identification of CKD status. It is possible that the relatively lower prevalence of CKD listing among those patients with higher eGFR_creat_ represents PCP discomfort with labeling persons with a disease when the eGFR_creat_ is close to guideline definition cut-points.

Finally, we found that CKD awareness, defined as CKD on the problem list, was significantly associated with higher rates of testing for albuminuria and CKD complications. It was also associated with higher prevalence of ACEi/ARB and statin prescription. These findings highlight improving CKD awareness as a “low hanging fruit” actionable gap in CKD care. Thus, pragmatic trials that include interventions to improve CKD awareness have the potential to improve some important processes of care.

### Strengths and limitations

We were able replicate an EHR CKD registry using previously described methods [19, 22]. A strength of our study was the selection of patients recently seen in primary care and with a recent “qualifying” eGFR_creat_ measurement. Our study also has several limitations. The observational study design and cross-sectional statistical methods makes it impossible to attribute causality. We reviewed a relatively small number of charts; however, we were about to demonstrate that our chart-review sample was representative of the larger cohort. We also cannot definitively conclude that the findings presented represent a primary care provider’s awareness of a patient’s CKD status. These findings are specific to EHR systems built using a problem list linked to each patient, and might not be generalizable to EHR systems without this architecture. However, it’s we expect discrepancies would still exist between an eGFR-defined CKD and the corresponding diagnosis codes. The lack of a problem-list-documented CKD diagnosis does not necessarily mean a provider was unaware of their patient’s CKD. We did not measure any physician behaviors or professional characteristics (e.g., workflow practices, time spent in clinical practice, medical practice team organization, etc.) that might influence EHR problem list use and guideline concordant processes of care. We considered including lab-defined CKD using a dipstick proteinuria, but decided against it because we felt this would create a bias by indication because the majority of the patients receiving proteinuria testing are diabetic. However, we did find good evidence that listing CKD on the problem list was associated with guideline-concordant care.

## Conclusions

Performing secondary analyses of EHR data to explore associations between patient characteristics, clinical measurements, delivery of care, and CKD may be useful and appropriate for hypothesis generation or risk prediction. Given the higher likelihood of CKD problem listing with lower eGFR_creat_, it may be particularly useful in studies of patients with more advanced disease. However, if the nature of the investigation is to identify patients with CKD who are most likely to benefit from an intervention, researchers should expect that CKD will need to be confirmed.

## Additional files

Additional file 1: Appendix **Table S1.** Diagnosis Codes for Selected Comorbid Conditions. ICD9 codes used to identify problem list diagnoses of patients. (XLSX 9 kb)

Additional file 2: Appendix **Table S2.** Characteristics of primary care patients with CKD based on eGFR (*n* = 1767). Patient characteristics stratified by their eGFR values. (XLSX 16 kb)

Additional file 3: Appendix **Table S3.** Patients with Documented Nephrology Consultations (*n* = 389). Logistic regression models for Patients who had a nephrology consultation in their electronic health record. (XLSX 14 kb)

Additional file 4: Appendix **Table S4.** Patients without Documented Nephrology Consultations (*n* = 1377). Logistic regression models for Patients who had a nephrology consultation in their electronic health record. (XLSX 14 kb)

## Abbreviations

ACE/ARB: Renin-angiotensin system
CKD: Chronic kidney disease
eGFR: Estimated glomerular filtration rate
eGFR_creat_: Estimated filtration rate by creatinine
EHR: Electronic health record
eMERGE: Electronic Medical Records and Genomics
ESRD: End stage renal disease
GIM: General internal medicine
KDIGO: Kidney disease improving global outcomes
PCP: Primary care physician
UCSF: University of California, San Francisco
